# Spontaneous Complete Uterine Rupture With Fetal Demise at Term Following Laparoscopic Myomectomy: A Case Report

**DOI:** 10.1002/ccr3.73188

**Published:** 2026-07-17

**Authors:** Xuejing Liu, Haiyan Liu, Lei Chen, Yuemei Zhang, Yingnuo Liu, Xiaoxiao Ni, Hongqing Jiang

**Affiliations:** ^1^ Department of Gynecology and Obstetrics Haidian Maternal and Child Health Hospital Beijing China

**Keywords:** diagnosis, intra‐abdominal hemorrhage, laparoscopic myomectomy, pregnancy, uterine rupture

## Abstract

Uterine rupture, a life‐threatening emergency in late pregnancy or labor, has been increasingly reported post‐laparoscopic myomectomy (LM). We report a case of spontaneous rupture with fetal demise managed surgically. This case highlights the diagnostic challenge in patients with LM history, emphasizing suspicion for rupture without classic symptoms for prompt intervention to optimize outcomes.

## Key Clinical Message

Uterine rupture, a severe complication endangering the mother and the fetus, typically occurs in late pregnancy or during delivery. Early clinical recognition and expeditious surgical intervention are critical to reduce the risk of catastrophic maternal and neonatal outcomes.

## 1 Introduction

Uterine rupture, defined as a complete separation of all layers of the uterine wall, is a serious obstetric complication associated with significant maternal and perinatal morbidity and mortality [1]. Following the revision of China's fertility policy, the uterine rupture rate has increased from 0.05% to 0.22%, associated with a 17.9‐fold increase in the incidence of critical maternal conditions and a 4.1‐fold elevation in the risk of stillbirth [2]. A scarred uterus, most commonly resulting from a previous cesarean section, is the predominant risk factor [3]; however, uterine rupture following myomectomy, particularly laparoscopic myomectomy (LM), is an increasingly recognized entity due to the rising prevalence of minimally invasive uterine surgery [4]. The reported incidence of uterine rupture after LM ranges from 0.5% to 1.0%; this risk is influenced by factors such as the number, size, location, depth, and the interval between surgery and conception [5]. However, diagnosing uterine rupture in late pregnancy presents a significant diagnostic challenge due to its variable and nonspecific clinical manifestations [6]. Delayed diagnosis may result in life‐threatening complications, including massive intra‐abdominal hemorrhage, maternal hypovolemic shock, fetal demise, and an increased likelihood of requiring emergency hysterectomy [7]. This case report holds significant clinical value as it describes a 33‐year‐old primigravida at 38^+3^ weeks of gestation, who had undergone a prior LM for a 6 × 6 × 5 cm anterior uterine intramural myoma. She presented with irregular abdominal pain and subsequently experienced a complete uterine rupture leading to fetal demise. This report aimed to enhance early identification, facilitate a rapid multidisciplinary response, and ultimately prevent adverse maternal and neonatal outcomes. This case has obtained the patient's written informed consent.

## 2 Case History

The patient was a 33‐year‐old primipara who had undergone laparoscopic myomectomy (LM) 12 months before conception. An intramural myoma 6 × 6 × 5 cm was located on the anterior wall of the uterus. The uterine cavity was not entered, and the uterine incision was closed in two layers with absorbable continuous suture. Electrocoagulation was used to incise the uterine serosa and myometrium, and the overall surgical procedure was not complicated. The patient conceived spontaneously 12 months later. Prenatal care was initiated at 12^+1^ weeks, with routine checkups throughout the pregnancy. Her prenatal screening tests and ultrasound examinations were unremarkable during the entire pregnancy. The patient presented with irregular abdominal pain starting at 01:00 on April 13, 2026, occurring every 5 min–10 min, without vaginal bleeding or fluid leakage. At 04:34, she was admitted to the emergency department, where Doppler fetal heart rate monitoring revealed a rate of 121 bpm. At approximately 05:18, she developed severe, continuous abdominal pulling pain that progressively worsened.

Upon admission, the patient's vital signs were as follows: temperature of 36.2°C, pulse of 120 beats per minute, respiratory rate of 19 breaths per minute, and blood pressure of 90/60 mmHg. She appeared pale and had cold, clammy skin. Abdominal examination revealed generalized tenderness and rebound tenderness. Fetal heart sounds were absent, uterine contractions were not obvious, and the uterine contour was indistinct. Significantly lower abdominal tenderness was noted. The fetal presentation was floating, unclear on palpation, and located between the umbilicus and pubis.

## 3 Differential Diagnosis, Investigations, and Treatment

A bedside emergency ultrasound indicated the following findings: (1) At 38 weeks of gestation, a single fetus was observed with no detectable fetal heart activity; (2) a discontinuity in the uterine myometrium, approximately 5.2 cm wide, communicating with the pelvic cavity was observed, containing heterogeneous echoes measuring about 5.0 × 5.2 cm; (3) an uneven echo was observed in the uterine cavity, connected to the posterior inferior margin of the placenta, with a range of approximately 11.4 × 6.1 cm, and no obvious blood flow signal was detected within it; (4) free fluid was noted in the abdominal cavity, with a depth of 3.1 cm in the right lower quadrant and 5.0 cm in the pelvis. Considering the patient's medical history, physical examination, and ultrasound results, the diagnosis of uterine rupture is clear. Immediate management involved establishing two intravenous lines for fluid resuscitation, continuous ECG monitoring, oxygen administration, and organizing a multidisciplinary team including the obstetrics, the anesthesiology department, the laboratory, the blood transfusion, and other disciplines for an urgent laparotomy exploration.

**FIGURE 1:**
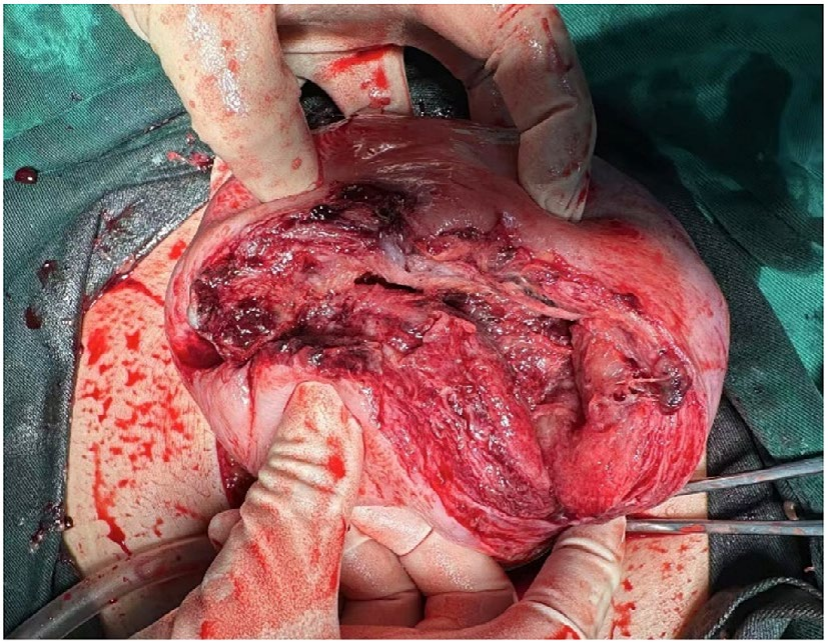
Uterine rupture with a “7”‐shaped configuration (approximately 10 cm transversely and 8 cm longitudinally) located on the anterior uterine wall.

**FIGURE 2:**
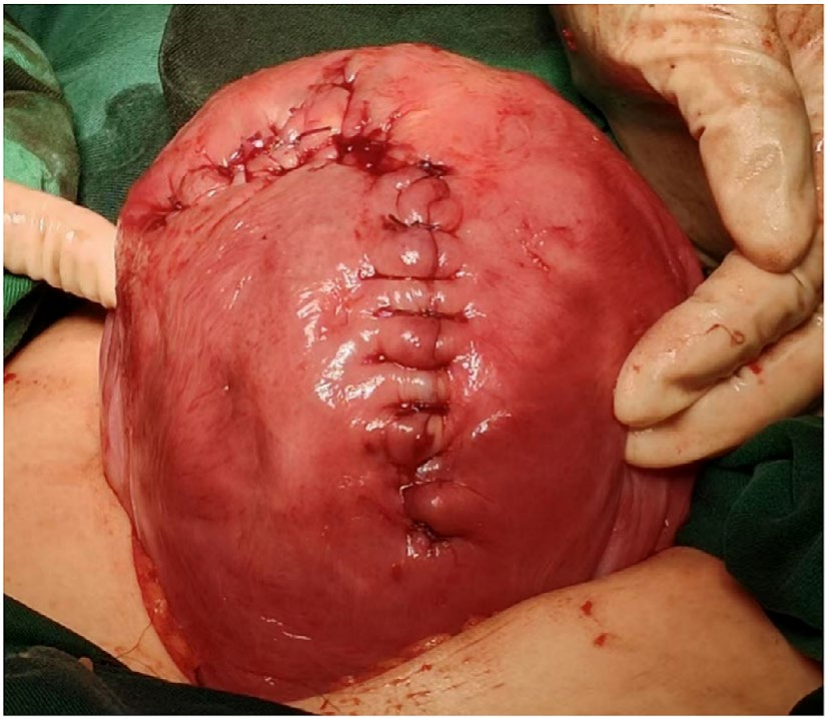
Uterine rupture repair with triple‐layer suture using absorbable suture.

The operation commenced at 06:15 under general anesthesia with tracheal intubation. An accumulation of dark red blood measuring approximately 2300 mL was observed in the abdominal cavity. A stillborn female infant weighing 3430 g and the intact placenta were delivered. A complete, fresh rupture with a “7”‐shaped configuration (approximately 10 cm transversely and 8 cm longitudinally) was observed at the site of the previous myomectomy scar on the anterior uterine wall (Figure 1). No active bleeding was noted at the rupture site; the posterior wall and the lower uterine segment were intact, and no hematoma was present in the parametrial area. Therefore, after obtaining informed consent from the family, a uterine rupture repair surgery was performed, with triple‐layer suture using absorbable suture (Figure 2). The operation was successfully concluded at 08:50. The endotracheal tube was removed at 09:33, and the patient was transferred to the ward at 10:50 with supplemental oxygen via face mask at 5 L/min and received symptomatic supportive treatment. Throughout the procedure, the patient received 8 units of alloleukocytic suspended red blood cells, 800 mL of fresh frozen plasma, along with other fluids totaling 5800 mL. Abdominal hemorrhage amounted to 2300 mL, intraoperative blood loss amounted to 200 mL, and urine output measured 2000 mL, for a total of 4500 mL.

## 4 Conclusion and Results

Placental pathological examination revealed marked dilation and congestion of the chorionic plate vessels with thrombosis, and morphological changes consistent with both maternal and fetal vascular malperfusion. The patient recovered well postoperatively and was discharged 8 days after surgery in a stable condition.

## 5 Discussion

Uterine rupture following myomectomy is a catastrophic obstetric emergency. The risk is significantly higher after the laparoscopic procedure (approximately 1%) compared than that following laparotomy (0.25%–0.42%) [8]. At present, LM has become a preferred surgical approach for uterine myoma due to a shorter hospital stay, decreased postoperative pain, and increased patient satisfaction [9]. However, concerns persist regarding the integrity of the uterine scar in subsequent pregnancies and the potential for uterine rupture. The case concerned a 33‐year‐old primigravida who underwent LM for a 6 × 6 × 5 cm anterior uterine intramural myoma in July 2024. She conceived 1 year postoperatively. The subsequent uterine rupture presented as a complete dehiscence at the prior surgical scar, resulting in a “7”‐shaped defect measuring approximately 10 cm in transverse and 8 cm in longitudinal dimension. The underlying etiology may involve iatrogenic thermal injury from widespread electrocautery application (leading to local tissue necrosis), limitations in surgical closure technique, and the added risk of infection—all of which can compromise scar integrity [10].

Furthermore, uterine myoma scars are particularly susceptible to rupture due to the relative thinness of the myometrium in this region, thereby increasing the risk of dehiscence under the mechanical stress of a progressing pregnancy [11]. A systematic review and meta‐analysis reported that post‐myomectomy uterine ruptures occur at a mean gestational age of approximately 34 weeks, with LM associated with rupture at a mean of 35^+6^ weeks [12]. Similarly, a case series of three patients described ruptures at 22^+4^, 31, and 36^+3^ weeks of gestation [13]. The timing of rupture at 38 weeks emphasizes that close monitoring must be maintained until delivery.

The typical clinical manifestations of uterine rupture consist of severe abdominal pain, vaginal bleeding, and fetal distress [12]. This clinical diagnosis typically involves a combination of the patient's clinical manifestations and imaging examinations [1]. However, this case illustrates an atypical presentation of uterine rupture, in which irregular abdominal pain was mistaken for prodromal labor, leading to a diagnostic delay. Terrinoni et al. reported a case of atypical uterine rupture discovered during an elective cesarean section at 35 weeks in a woman with a history of LM, presenting with scar dehiscence and partial fetal limb entrapment, but without classic acute pain or hemorrhagic signs [14]. These serve as a critical reminder that a high index of suspicion for rupture is mandatory in any pregnant woman with a history of LM, particularly when she presents with any form of abdominal pain in late pregnancy. Moreover, this case reinforces the need for individualized risk assessment and intensified antenatal surveillance, including serial ultrasound evaluation of the myometrial scar in late pregnancy, for all women with a history of LM [15].

The management of complete uterine rupture mandates immediate surgery and activation of a multidisciplinary team [16]. Uterine repair is preferred for women wishing to preserve fertility, provided the defect is repairable and the patient is stable. However, total hysterectomy may be indicated based on intraoperative findings and maternal stability, particularly in cases of extensive or irregular defects, severe infection, critical vascular involvement, or when repair is deemed technically inadequate [17]. Potential sequelae include infection, adhesions, and future uterine scar weakness. Therefore, early recognition and a swift, coordinated multidisciplinary response are paramount to optimizing maternal and neonatal outcomes.

## 6 Conclusion

This case mandates heightened vigilance for uterine rupture in all pregnant patients with prior LM, even amid nonspecific symptoms, and necessitates a rapid, multidisciplinary response to optimize maternal outcomes. We emphasize the necessity of late‐pregnancy ultrasound surveillance to assess myometrial continuity. For future pregnancies, risk reduction hinges on preconception planning, close monitoring, and elective cesarean section. The insights from this single case, while instructive, warrant validation in larger cohorts to establish generalizability.

## Author Contributions

**Hongqing Jiang:** supervision, writing – review and editing. **Xiaoxiao Ni:** investigation. **Yingnuo Liu:** investigation. **Yuemei Zhang:** conceptualization. **Lei Chen:** writing – review and editing. **Xuejing Liu:** conceptualization, writing – original draft. **Haiyan Liu:** conceptualization.

## Funding

The authors have nothing to report.

## Consent

Written informed consent was obtained from the patient for publication of this case report and accompanying images.

## Conflicts of Interest

The authors declare no conflicts of interest.

## Data Availability

The data that support the findings of this study are available on request from the corresponding author. The data are not publicly available due to privacy or ethical restrictions.
